# Supplementary material for: E-Professionalism among Dental Students from Malaysia and Finland
Source: Int J Environ Res Public Health. 2022 Mar 9;19(6):3234. doi: 10.3390/ijerph19063234 (PMC8949338; doi:10.3390/ijerph19063234)
Supplement: Supplementary file 1 [file ijerph-19-03234-s001.zip › Final Questionnaire.pdf]

Questionnaire on Social Media usage by Dental Students

1. What dental school are you at?
    1. University of Helsinki
    2. University of Oulu
    3. Manipal University College Malaysia
    4. Faculty of Dentistry, University Malaya

2. What is your gender?
    5. Male
    6. Female

3. What is your age?
    1. 20 or younger
    2. 21-23
    3. 24-26
    4. 27-29
    5. 30-35
    6. 35 and above

4. How do you identify yourself? (In the Finnish version, this wase replaced by the following text (in Finnish): The question is related to ethnic background and does not concern Finnish students)
    1. Malay
    2. Chinese
    3. Indian
    4. Other Bumiputera
    5. Other (please specify) ………………………..

5. What year of dental school are you currently in?
    1. First
    2. Second
    3. Third
    4. Fourth
    5. Fifth
    6. Other (please specify) …………………

6. How familiar are you with each of the following social media services?

	Never heard of it	Heard of it; not sure of its purpose	Familiar with its purpose	Very familiar with its purpose
○ Facebook				
○ Twitter				
○ LinkedIn				
○ YouTube				
○ Google+				
○ Pinterest				
○ Tumblr				
○ Instagram				
○ WhatsApp				
○ Snapchat				
○ Jodel				
○ TikTok				
○ Telegram				
○ WeChat				
○ Weibo				

If there are other social media services with which you are familiar, please list and rate your familiarity here.

…………………………………………………………………………………………………….…………..…

7. How competent are you at using each of the following social media services?

	Not at all	Beginner	Competent	Highly competent
○ Facebook				
○ Twitter				
○ LinkedIn				
○ YouTube				
○ Google+				
○ Pinterest				
○ Tumblr				
○ Instagram				
○ WhatsApp				
○ Snapchat				
○ Jodel				
○ TikTok				
○ Telegram				
○ WeChat				
○ Weibo				

If there are other social media services that you use, please list and rate your competence here:

……………………………………………………………………………………..……………………

8. How often do you use each of the following social media services?

	Never	Occasionally (not usually more than once a month)	Regularly (not daily but at least weekly)	Frequently (daily)	Very frequently (several times a day
○ Facebook					
○ Twitter					
○ LinkedIn					
○ YouTube					
○ Google+					
○ Pinterest					
○ Tumblr					
○ Instagram					
○ WhatsApp					
○ Snapchat					
○ Jodel					
○ TikTok					
○ Telegram					
○ WeChat					
○ Weibo					

If there are other social media services that you use, please list and rate how often you use them here:
…………………………………………………………………………………………....…………..

9. How important is each of the following factors in encouraging you to use social media?

	Not at all important	Somewhat important	Very important	I don't use social media
To stay in touch with current friends and family members				
To connect with old friends, I have lost touch with				
To connect around a shared hobby				
To communicate about issues relating to dental training				

10. How much do the following reasons prevent you from using social media?

	Not at all	Somewhat	Very much
Lack of knowledge			
Lack of time			
Lack of interest			
Lack of perceived value			
Concern about harm to professional image			

Other (please specify): ……………………………………………………………………………….………..

11. Approximately how many hours **A WEEK** do you spend using social media?

- Do not use)
- Less than 5 hours
- 6-10 hours
- 11-15 hours
- 16-20 hours
- More than 20 hours

12. Approximately how many hours **A WEEK** do you spend using the following social media services as **part of your dental education**?

	Do not use	Less than 1 hour	1-5 hours	6-10 hours	11-15 hours	16-20 hours	More than 20 hours
o Facebook							
o Twitter							
o Linked In							
o YouTube							
o Google+							
o Pinterest							
o Tumblr							
o Instagram							
o Whatsapp							
o Snap chat							
o Jodel							
o Tik Tok							
o Telegram							
o WeChat							
o Weibo							

13. How often do you monitor your online presence (e.g., Google yourself)?

- o Never
- o Occasionally (not usually more than once a month)
- o Regularly (not daily but at least weekly)
- o Frequently (daily)
- o Very frequently (several times a day)

14. How often do you check social media for photos of yourself?

- o Never
- o Occasionally (not usually more than once a month)
- o Regularly (not daily, but at least weekly)
- o Frequently (daily)
- o Very frequently (several times a day)

15. How important is each of the following reasons for monitoring your online presence?

	Not at all important	Somewhat important	Very important
To ensure that posted information is accurate			
To ensure that posted information is complete			
To ensure that posted information is professional			

Other (please specify): ………………………………………………………………………………

16. What action have you taken if you find information that you believe should not be publicly available? Please check all answers that apply.

1. Deleted people from my "friends" list
2. Stopped "following" people
3. Deleted comments made by others on my profile
4. Removed my name from photos that were tagged to identify me
5. I have not taken action.

Other (please specify): ………………………………………………………………………..

17. Have you ever found that your online presence is: Please check all answers that apply.

1. Inaccurate
2. Incomplete
3. Unprofessional
4. Absent

Other (please specify): ……………………………………………………………………..…..

18. I use privacy settings.

- Yes
- No

19. I have accepted invitation(s) from patients to be "friends."

- Yes
- No

20. I have invited patients to be "friends."

- Yes
- No

21. I have accepted invitations by faculty to be "friends."

- Yes
- No

22. I have invited faculty to be "friends."

- Yes
- No

23. I have Googled faculty members.

- Yes
- No

24. Which of the following content have you posted online yourself?

Please check all answers that apply

1. None
2. Unidentifiable patient information
3. Identifiable patient information
4. Obscene language
5. Discriminatory language
6. Depiction of intoxication
7. Sexually suggestive material
8. Items I thought were initially appropriate, but for various reasons later took down.

Other (please specify): ……………………………………………..……………….

25. Which of the following types of information have you seen posted online by a classmate?

Please check all answers that apply.

1. None
2. Unidentifiable patient information
3. Identifiable patient information
4. Use of profanity
5. Depiction of intoxication
6. Sexually suggestive material
7. Items I found objectionable but did not discuss with my classmate
8. Items I found objectionable and did discuss with my classmate

Other (please specify): ……………………………………………………………….

26. List the social media services you are aware of that provide benefit to patients.

…………………………………………………………………………………………

27. When I become a dentist, I intend to use social media to interact with my patients.

- o Yes
- o No
- o Undecided

28. What concerns do you have about social media use?

	Not at all important	Somewhat important	Very important
Public perceptions of unprofessional behavior by me			
Family perceptions of unprofessional behavior by me			
Public perceptions of unprofessional behavior by my colleagues			
Public perceptions of my dental school			
Public perceptions of the dental profession			
Violations of patient confidentiality			
Posting of inaccurate Dental related information for patients			

Other (please specify) …………………………………………………………………

29. To what extent do you agree or disagree with the following statement?

Patients use social media to obtain dental information.

- Strongly agree
- Agree
- Disagree
- Strongly disagree

30. To what extent do you agree or disagree with the following statement?

The benefits of social media use in dentistry outweigh its risks.

- Strongly agree
- Agree
- Disagree
- Strongly disagree

31. To what extent do you agree or disagree with the following statement?

As a student of dentistry, it is my obligation to keep current on social media use.

- Strongly agree
- Agree
- Disagree
- Strongly disagree

32. To what extent do you agree or disagree with the following statement?

Guiding patients online is a new responsibility for dentists in the digital age.

- Strongly agree
- Agree
- Disagree
- Strongly disagree

33. Your personal data can be made accessible online without your deliberate intervention.

- True
- False

34. My university has a policy on social media usage.

- True
- False
- Do not know

35. Which dentally related websites or blogs do you visit regularly?

…………………………………………………………………………….

36. Please add any additional comments about social media and dental professionalism here:

…………………………………………………………………………………………..……………..

37. What additional information would you like to learn about social media?

……………………………………………………………………………….…..
