# Supplementary material for: E-Professionalism among Dental Students from Malaysia and Finland
Source: Int J Environ Res Public Health. 2022 Mar 9;19(6):3234. doi: 10.3390/ijerph19063234 (PMC8949338; doi:10.3390/ijerph19063234)
Supplement: Supplementary file 1 [file ijerph-19-03234-s001.zip › PIS.pdf]

PARTICIPANT INFORMATION SHEET

Please read the following information carefully. Do not hesitate to contact the investigator for any questions you may have.

Study Title

Social media usage and E-professionalism among dental undergraduate students - a comparative study

Introduction

Social media is useful not only for marketing and recruitment, but also for education and networking. Studies in health professionals' education have found benefits in use of social media tools in clinical education. While these technologies have helped the students in garnering knowledge and skills related to their profession, potential risks have been identified, related to social media use such as creation of a negative digital footprint, compromised professional relationships, litigation due to misconduct, and detrimental physiological effects for users based on dopamine release. The curriculum, teaching and learning approaches may be different between Asian and European countries and may have an influence on the use of social media in the dental studies. There are also significant cultural differences which may affect the use of different applications and technologies.

What is the purpose of this study?

This is a comparative study between dental students in the academic year 2020-21 from two different countries (Malaysia and Finland) to help understand the extent and nature of social media use among undergraduate dental students.

What are the procedures to be followed?

Once you have read the participant information sheet, you will be required to give consent online by ticking the required box. You will be directed to the online questionnaire consists of 37 items with open ended and closed ended questions. It will take about 10 minutes to answer the questionnaire. All questions need to be answered.

Who should not enter the study?

If you are not a undergraduate student at the Faculty of Dentistry, MUCM in the academic year 2020-21, please refrain from proceeding with the questionnaire

What will be the benefits of the study:

(a) *To participant?*
The results of this study will help understand the pattern of social media usage among dental students. It will benefit the educators with regards to ethics and professionalism in social media.

(b) *To the investigator?*
This study will provide suggestions for social media use training in the dental curriculum.

What are the possible risks / complications / adverse effects that may happen?

There are no risks/ complications/ adverse effects by participating in this survey. All data will remain anonymous and will not be used for purposes other than for research.

Can I refuse to take part in the study?
Your participation is totally voluntary. You need not have to explain why you prefer not to take part in the study and it will not affect your student candidature.

Who shall I contact if I have additional questions during the course of the study?

(1) Investigator's Name: Prof. Dr. Eswara Uma
Contact no: 06 2896662 extn 3820
Address: Department of Paediatric Dentistry, Faculty of Dentistry, MUCM, Melaka
Email address: eswara.uma@manipal.edu.my

(2) Investigator's Name: A/P Dr Eby Varghese
Contact no.: 062896662 extn 3831
Address: Department of Paediatric Dentistry, Faculty of Dentistry, MUCM, Melaka
Email address: eby.varghese@manipal.edu.my

INFORMED CONSENT

Social media usage and E-professionalism among dental undergraduate students - a comparative study

By submitting the complete survey, I confirm the following:

- I have been given written information for the above study and have read and understood the information given.
- I have had sufficient time to consider participation in the study and have had the opportunity to ask questions and all my questions have been answered satisfactorily.
- I understand that my participation is voluntary and I can at anytime freely withdraw from the study. I understand the risks and benefits, and I freely give my informed consent to participate under the conditions stated. I understand that I must follow the investigator's instructions related to my participation in the study.

I agree ☐ I disagree ☐
